# Reverse Triggered Breath during Pressure Support Ventilation and Neurally Adjusted Ventilatory Assist at Increasing Propofol Infusion

**DOI:** 10.3390/jcm12144857

**Published:** 2023-07-24

**Authors:** Federico Longhini, Rachele Simonte, Rosanna Vaschetto, Paolo Navalesi, Gianmaria Cammarota

**Affiliations:** 1 Anesthesia and Intensive Care, Department of Medical and Surgical Sciences, “Magna Graecia” University, 88100 Catanzaro, Italy; 2 Division of Anesthesia, Analgesia and Intensive Care, Department of Medicine and Surgery, Hospital S. Maria della Misericordia, University of Perugia, 06123 Perugia, Italy; 3 Anesthesia and Intensive Care, Department of Translational Medicine, Eastern Piedmont University, 28100 Novara, Italy; 4 Anesthesia and Intensive Care, Padua Hospital, Department of Medicine—DIMED, University of Padua, 35128 Padova, Italy; paolo.navalesi@unipd.it

**Keywords:** pressure support ventilation, neurally adjusted ventilatory assist, propofol, patient-ventilator asynchrony, sedation, reverse triggered breath

## Abstract

Background: Reverse triggered breath (RTB) has been extensively described during assisted-controlled modes of ventilation. We aimed to assess whether RTB occurs during Pressure Support Ventilation (PSV) and Neurally Adjusted Ventilatory Assist (NAVA) at varying depths of propofol sedation. Methods: This is a retrospective analysis of a prospective crossover randomized controlled trial conducted in an Intensive Care Unit (ICU) of a university hospital. Fourteen intubated patients for acute respiratory failure received six trials of 25 minutes randomly applying PSV and NAVA at three different propofol infusions: awake, light, and deep sedation. We assessed the occurrence of RTBs at each protocol step. The incidence level of RTBs was determined through the RTB index, which was calculated by dividing RTBs by the total number of breaths triggered and not triggered. Results: RTBs occurred during both PSV and NAVA. The RTB index was greater during PSV than during NAVA at mild (1.5 [0.0; 5.3]% vs. 0.6 [0.0; 1.1]%) and deep (5.9 [0.7; 9.0]% vs. 1.7 [0.9; 3.5]%) sedation. Conclusions: RTB occurs in patients undergoing assisted mechanical ventilation. The level of propofol sedation and the mode of ventilation may influence the incidence of RTBs.

## 1. Introduction

Patient-–ventilator asynchrony (PVA) refers to the lack of coordination between the patient’s efforts and ventilator assistance. It affects approximately 25% of patients receiving assisted mechanical ventilation, and is seemingly linked to adverse patient outcomes [1,2].

Reverse triggered breath (RTB) has been more recently described as a patient’s inspiration triggered by ventilator insufflation during assisted-controlled modes [3]. An established fixed repetitive temporal relationship between ventilator insufflation and the neural respiratory cycle (i.e., respiratory entrainment) characterizes RTB [3]. In addition, if the respiratory effort is strong enough a second mechanical insufflation can be triggered, resulting in breath stacking (BS) [3].

Sedatives and analgesic drugs change the respiratory drive and/or timing [4,5,6]. Propofol, an increasingly utilized short-acting sedative-hypnotic agent in Intensive Care Units (ICUs), is recommended for mechanically ventilated ICU patients. It has been found to be at least as effective as midazolam, with the added benefits of a more rapid and predictable time of emergence and a shorter interval to extubation [4]. As no previous study has examined the impact of this sedative on patients receiving partial ventilatory assistance, we undertook a randomized cross-over physiologic study. Our aim was to assess the effects of three steady-state propofol concentrations representing different levels of sedation (wakefulness, light, and deep sedation) on various aspects such as ineffective triggers, gas exchange, breathing pattern, respiratory drive, and timing. This investigation specifically focused on patients with acute respiratory failure (ARF) who were receiving partial ventilatory assistance delivered by either Pressure Support Ventilation (PSV) or Neurally Adjusted Ventilatory Assist (NAVA) [4]. We found that deep sedation with propofol markedly reduced the respiratory drive and increased the incidence of ineffective efforts during PSV, but not during NAVA. [4]. Lower doses of propofol which induce milder sedation, as well as remifentanil or dexmedetomidine at any dosage, do not have any effects on the occurrence of PVA [4,5,6].

To date, RTB has been reported only during assist-controlled modes and more frequently during deep sedation [3]. No studies have investigated the occurrence of RTB during assisted modalities. Indeed, auto-triggered breaths are mechanical (i.e., ventilator) insufflations triggered by airway pressure (Paw) or flow signal disturbance produced by condensed water in the ventilator circuit [7], copious tracheobronchial secretions [7], cardiac oscillations [8] or air-leaks [9]. Auto-triggered breaths have the potential to function as mandatory ventilator insufflations, which, in turn can elicit patient efforts in a reverse manner. Basing our hypothesis on this, in this preliminary and descriptive investigation we have retrospectively analyzed the waveforms of our previous study [4] to assess whether RTB occurs during PSV and NAVA. In addition, we have verified the effect of different levels of propofol sedation may on the occurrence of RTB.

## 2. Materials and Methods

Recordings were obtained from the ICU of the University Hospital of Novara (Italy) between 1st August 2008 and 28th February 2009 following the principles outlined in the Declaration of Helsinki. The study received approval from the local Ethics Committee (approval no. 72 on 27th August 2007). The primary objective of the study was to evaluate the effects of three levels of propofol sedation on the respiratory drive, timing, and patient–ventilator asynchrony during both Pressure Support Ventilation (PSV) and Neurally Adjusted Ventilatory Assist (NAVA) [4]. We included fourteen intubated patients with central venous and arterial indwelling catheters who were receiving partial ventilatory support for a maximum duration of 48 h. These patients had been administered short-acting sedative agents, specifically propofol and/or remifentanil, and had a Glasgow Coma Scale score higher than 10 when sedation was discontinued [4].

Patients were excluded from the study if they met any of the following criteria: (1) age below 18 years; (2) contraindications for the placement of an Electrical Activity of the Diaphragm (EAdi) catheter, such as esophageal varices, upper gastro-esophageal bleeding within the last 30 days, or gastro-esophageal surgery in the past 12 months; (3) hemodynamic instability despite adequate fluid volume (requiring epinephrine or vasopressin infusion, dopamine or dobutamine > 5 µg∙kg^−1^∙min^−1^, or norepinephrine > 0.1 µg∙kg^−1^∙min^−1^ to maintain mean arterial blood pressure > 60 mmHg); (4) core temperature exceeding 38 °C; (5) renal failure (blood creatinine ≥ 110 μmol/L); (6) pregnancy; (7) presence of major painful stimuli such as recent surgical wounds or traumatic injuries; (8) history of allergy to propofol components; (9) inability to maintain a tidal volume (VT) ≤ 8 mL/kg with a minimum inspiratory support of 8 cmH_2_O; (10) Positive End-Expiratory Pressure (PEEP) > 12 cmH_2_O and/or inspired oxygen fraction (FiO_2_) > 0.6; (11) prior propofol infusion exceeding 2 mg∙kg^−1^∙h^−1^ lasting 8 h or more or less than 2 mg∙kg^−1^∙h^−1^ for less than 8 h whenever propofol wash-out was not possible due to agitation (as defined by the Ramsay Sedation Scale),hypertension (arterial systolic pressure > 180 mmHg) and tachycardia (>125 bpm), or unbearable patient discomfort; or (12) inclusion in other research protocols [4].

Protocol discontinuation occurred in the event of: (1) hemodynamic instability (as defined in the aforementioned exclusion criteria; (2) agitation; or (3) inability to maintain arterial oxygen saturation (SpO_2_) ≥ 92% [4].

## 3. Study Protocol

We conducted mechanical ventilation using a Servo-I ventilator (Maquet Critical Care, Solna, Sweden), which is capable of delivering both PSV and NAVA. To obtain the EAdi we utilized a nasogastric feeding tube with an array of electrodes placed at its distal end (EAdi catheter, Maquet Critical Care, Solna, Sweden).

Propofol 2% was administered intravenously through a central vein using the DiprifusorTM (AstraZeneca, Macclesfield, Cheshire, UK) Target Controlled Infusion (TCI) system. This system utilizes an infusion pump (Terumo TE-372, Terumo Corporation, Ashitaka Factory, Tokyo, Japan) and incorporates the Marsh pharmacokinetic model. Rather than setting the drug infusion rate, the TCI system allows the user to input the patient’s age, ideal body weight, and the desired target blood concentration of propofol. The system then calculates and adjusts the infusion rate accordingly to achieve and maintain the specified target concentration throughout the administration [4]. The level of sedation was continuously monitored using the Bispectral index (BIS) provided by the BIS monitor (BIS© monitor, Covidien Medical, Boulder, CO, USA). This monitoring system delivers a numeric output on a scale of 0 (indicating absence of brain electrical activity) to 100 (representing a fully awake state), thereby indicating the patient’s level of consciousness [4].

After positioning the EAdi catheter while the patient was awake, we adjusted the inspiratory pressure support to achieve a tidal volume (VT) of 6–8 mL/kg during active inspiration. Throughout the study period, the PSV mode on the Servo-I ventilator was set to default inspiratory and expiratory trigger settings. The inspiratory trigger level was set at 5, which corresponds to 50% of the 2 L/min bias flow. The expiratory trigger setting was set to 30% of the peak inspiratory flow [4].

Additionally, the level of assistance during NAVA was determined while the patient was awake by matching both VT and EAdi as closely as possible to the corresponding values observed during PSV [4]. NAVA triggers were set to default settings. The neural inspiratory trigger was set at 0.5 μV for NAVA, while the expiratory trigger was fixed at 70% of the peak EAdi [4]. The FiO_2_ and PEEP were maintained at the same values used before patient enrollment, and were kept constant throughout the entire study period [4]. A 20 s apnea limit was set to initiate backup-controlled ventilation for safety reasons.

All patients received three levels of sedation: (1) no sedative infusion (patient awake); (2) deep sedation, achieved by setting the propofol target blood concentration to reach a BIS value of 40; and (3) light sedation, corresponding to half the propofol target blood concentration used to achieve BIS 40. Each patient underwent two 25 min trials in both PSV and NAVA at all three levels of sedation. The six trials were conducted in a random order determined by a computer-generated random sequence [4]. Notably, the 25 min trials started after at least 5 min of sedation level stabilization in order to avoid carryover effects between adjacent recordings.

## 4. Data Acquisition and Analysis

We recorded flow, Paw, and EAdi from the ventilator using dedicated software (NAVA Tracker V. 2.0, Maquet Critical Care, Sölna, Sweden) and performed manual offline analysis [4].

We classified all breaths as follows:

- Triggered breath: a mechanical (i.e., ventilator) insufflation triggered by a neural effort (i.e., a contraction of the diaphragm), defined by EAdi greater than 1 μV.
- Ineffective effort: a neural effort, as defined above, not followed by a ventilator pressurization.
- Auto-triggered breath: a mechanical insufflation without a negative deflection in Paw (i.e., not triggered by the patient) and with no neural effort.
- Double-triggered breath: two ventilator insufflations separated by a very short expiratory time (i.e., <30% of the mean inspiratory time) triggered by one patient’s effort.
- Reverse triggered breath (RTB): presence of a ventilator insufflation without a negative deflection in Paw followed by the initiation of a neural effort (i.e., increase in EAdi) greater than 1 μV [10]. BS phenomena were counted whenever they occurred.

We analyzed the entirety of the 25 min trials to look for all asynchronous events. We computed the RTB index (%) by dividing the number of RTBs by the total number of breaths, i.e., breaths triggered + not triggered.

## 5. Statistical Analysis

Due to the relatively small sample size of patients, data are presented as the median (25–75% interquartile range). We compared all continuous variables between different depths of sedation within each ventilatory mode. Repeated measures analysis of variance by rank was performed using nonparametric Friedman tests. Pair-wise comparisons were conducted using the Wilcoxon test, and the threshold for statistical significance was adjusted through Bonferroni correction for multiple comparisons. *P* values less than 0.017 were considered significant. For categorical data we utilized the Fisher exact test, and *p* values less than 0.05 were considered significant.

## 6. Results

We analyzed ventilator waveforms of fourteen patients. Mean ± Standard Deviation target propofol concentrations were 2.52 ± 0.71 μg/mL and 1.26 ± 0.35 μg/mL for deep and light sedation, respectively. The Ramsey Sedation Scale averaged 2.2 ± 0.4 for the awake state, 3.9 ± 1.3 for light sedation, and 6.0 ± 0.0 for deep sedation. PEEP and FiO_2_ were 8.4 ± 3.1 cmH_2_O and 0.42 ± 0.08, respectively. The levels of inspiratory assistance were 11.7 ± 3.3 cmH_2_O and 2.1 ± 1.6 cmH_2_O/µV for PSV and NAVA, respectively [4]. We have published data on the respiratory pattern and drive elsewhere [4].

Table 1 reports the median [IQR] of the absolute count of mechanical insufflations, neural efforts, and asynchronies observed per patient. Of note, all RTBs were sporadic without BS or respiratory entrainment phenomena.

**Figure 1:**
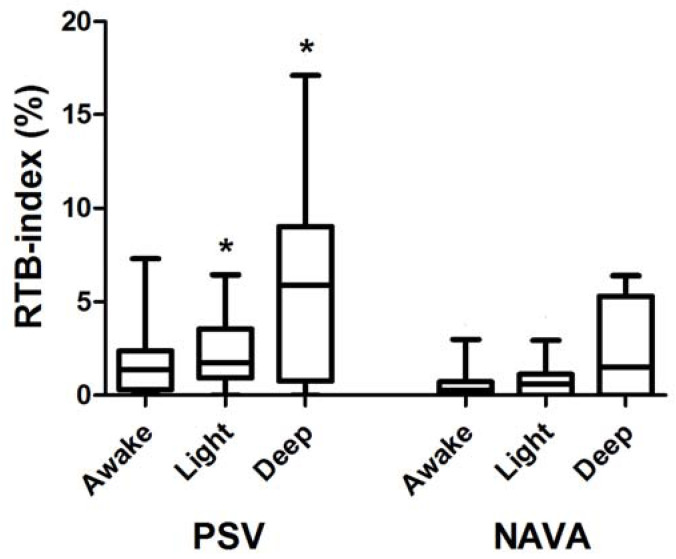
The figure depicts the RTB index during both PSV (left) and NAVA (right) at different propofol infusion rates (awake, light, and deep sedation). Data are shown in box-and-whisker plots. The bottom and top of the box show the 25th and 75th percentile; the horizontal band near the middle of the box is the median, and the ends of the whiskers represent the 10th and 90th percentile (see text for further explanation). * *p* < 0.017 between PSV and NAVA within the same level of sedation.

Figure 1 depicts the recorded RTB-index during both PSV (left) and NAVA (right) at different propofol infusion rates.

During PSV, the RTB index recorded while patients were awake (1.4 [0.3; 2.4]%) was not different from the corresponding values recorded during light sedation (1.7 [0.9; 3.5]%) or deep sedation (5.9 [0.7; 9.0]%). Similarly, during NAV the, RTB index recorded while patients were awake (0.2 [0.0; 0.7]%) was not different from the corresponding values recorded during light sedation (0.6 [0.0; 1.1]%) or deep sedation (1.5 [0.0; 5.3]%). The RTB index was not different between ventilatory modes during wakefulness (*p* = 0.032). In contrast, the RTB indexes during light (*p* = 0.004) and deep (*p* = 0.010) sedation were higher in PSV than in NAVA (Figure 1).

## 7. Discussion

Our study shows that RTBs occur in patients undergoing PSV and NAVA. Furthermore, the RTB index is higher at light and deep sedation during PSV than during NAVA.

To the best of our knowledge, this is the first study showing that RTBs appear during assisted modes of ventilation. Mandatory controlled breath inducing the diaphragm activation characterizes RTB. RTBs occur in acutely ill patients undergoing assist-controlled ventilation, sometimes with a repetitive pattern, i.e., entrainment [3].

In patients undergoing assisted modes of ventilation, a mechanical insufflation unrelated to a patient’s inspiratory activity is defined as an auto-triggered breath. In this case, pressurization is frequently initiated by alterations in Paw or flow caused by condensed water in the ventilator circuit [7], substantial tracheobronchial secretions [7], cardiac oscillations [8], or air leaks [9]. When an auto-triggered breath occurs during PSV, the ventilator delivers the set inspiratory pressure support to the patient’s airway. With auto-triggered breath during NAVA, the ventilator pressurizes 2 cm H_2_O of inspiratory pressure above PEEP based on the first-serve-first principle [11]. If an auto-triggered breath (acting as a mandatory breath) activates the diaphragm, an RTB occurs. It can be argued that such RTBs may have occurred due to apnea backup ventilation; however, it is important to note that apnea ventilation was not implemented during any of the analyzed recordings. This can be easily explained by the fact that, in contrast to remifentanil, propofol affects the respiratory drive rather than the timing [4,5].

We observed that the incidence of RTBs is higher under deep sedation during PSV. Previous studies have suggested that RTBs are more frequent in heavily sedated patients under assisted-controlled ventilation [3,12]. However, they can obscurely occur during wakefulness as well [13].

Interestingly, Mellado Artigas et al. recently showed that sedation levels did not differ between patients with low (<8%) or high (>8%) incidence of RTBs during assisted-controlled modes [10]. In addition, they showed that patients with a higher incidence of RTBs were more likely to be extubated within 24 h after recording [10]. Therefore, RTBs seem to occur during the transition phase between deep sedation and ventilator triggering and could represent the first step to recovering neural drive [10]. Of note, our patients should be considered to be in the recovering phase of the neural drive, given the inclusion criteria.

Deep sedation induced by propofol affects the output of the respiratory centers, leading to the suppression of the respiratory drive, while having minimal and insignificant effects on respiratory timing [4]. This effect was more pronounced during PSV than NAVA [4], partially explaining the different RTB indexes at light and deep sedation between modes of ventilation.

Of note, we did not observe any BS. BS has been described in patients with high respiratory drive during assisted/controlled modes of ventilation [14,15]. During the expiratory phase of the ventilator, the activation of the inspiratory muscles produces an eccentric contraction of the diaphragm [14]. The trigger of a second mechanical insufflation before complete exhalation results in an increased and injurious total tidal volume [15]. The reduction of the respiratory drive induced by propofol, together with the characteristics of our population, may be the reason that we did not observe any BS.

Although this is the first (and preliminary) study reporting the occurrence of RTB during PSV and NAVA and its incidence at different levels of propofol infusion, our results should be considered in the light of the following limitations. First, we detected RTBs by analyzing ventilator waveforms along with the EAdi as an adjunctive signal of our patients’ respiratory effort [10]. The detection of PVA solely through ventilator waveforms is difficult during both invasive [16] and non-invasive [17] ventilation. To overcome this limitation, we used an adjunctive signal (i.e., EAdi) considered necessary for this purpose [10,18]. However, EAdi detects only the diaphragm depolarization; the esophageal pressure would additionally sense accessory muscle contraction [19]. Hence, we cannot rule out the possibility that accessory inspiratory muscles might have initiated insufflations, as opposed to changes in Paw or flow, as occurs with auto-triggering [7,8,9]. In keeping with a previous study [10], we considered only ventilator insufflations without a drop in Paw and followed by the activation of the diaphragm as RTBs. Therefore, we reasonably excluded breaths triggered by accessory muscles, which would have generated a drop in Paw in case of their activation [10]. Second, we solely analyzed the effects of propofol on the occurrence of RTBs during both PSV and NAVA. Remifentanil can affect the breathing centers by prolonging the expiratory time without any effect on the respiratory drive or occurrence of asynchronies [5], while dexmedetomidine does not produce effects on respiratory effort or timing [6]. Consequently, based on the mechanisms of action and the observed effects on inspiratory muscles, it is reasonable to hypothesize that these drugs should not significantly impact the occurrence of RTBs as the infusion dose increases. Nevertheless, in order to establish the validity of this hypothesis and draw definitive conclusions, dedicated studies specifically designed to assess the impact of these drugs on RTBs are essential. Such research endeavors will provide valuable insights into the relationship between sedative agents and RTBs, contributing to a deeper understanding of assisted ventilation and potentially guiding clinical practice to optimize patient outcomes. Third, RTBs may activate inspiratory muscles other than the diaphragm [20]. To assess activation of the pectoralis or intercostal muscles, dedicated electromyography or ultrasonography is required [19,20]; unfortunately, we lack this signal to assess extra-diaphragm inspiratory muscles. This aspect holds promising potential for future investigations, and presents an intriguing avenue to explore. The distinct effects of both PSV and NAVA in diminishing the activation of extra-diaphragm inspiratory muscles as inspiratory support increases offer an area of study worth delving into [21]. Understanding these differences could shed light on the optimal utilization of ventilation modes and aid in refining respiratory support strategies. The knowledge gained from such research endeavors has the potential to enhance patient care and improve outcomes in the field of assisted ventilation.

In conclusion, RTBs occur during assisted modes of ventilation. Propofol infusion increases the incidence of RTBs during PSV as compared to NAVA. Further studies are necessary in order to delve deeper into the neural mechanisms underlying RTBs during assisted modes of ventilation. Moreover, additional research is needed to explore the impact of different sedatives on the occurrence of RTBs and to investigate their potential role in exacerbating or mitigating these asynchronies. There is a critical need to identify specific patient subgroups that might be at a higher risk of experiencing RTBs in order to aid in developing targeted interventions and personalized management strategies to optimize ventilatory support and enhance patient outcomes.

## Figures and Tables

**Table 1:** Absolute count of breaths and asynchronies analyzed from the waveforms.

	PSV			NAVA		
	Awake	Light	Deep	Awake	Light	Deep
Mechanical insufflations (n)	500 [400; 570]	457 [341; 530]	414 [252; 625]	536 [410; 594]	527 [398; 662]	552 [352; 696]
Neural efforts (n)	495 [398; 619]	442 [375; 523]	463 [251; 748]	536 [410; 594]	527 [398; 662]	552 [352; 696]
Ineffective Efforts (n)	7 [4; 9]	6 [2; 9]	11 [1; 126]	0 [0; 0]	0 [0; 0]	0 [0; 0]
Auto-Triggered breaths (n)	4 [2; 17]	8 [0; 17]	0 [1; 23]	0 [0; 0]	0 [0; 0]	0 [0; 0]
Double Ttriggered breaths (n)	0 [0; 1]	0 [0; 0]	0 [0; 0]	0 [0; 0]	0 [0; 0]	0 [0; 0]
RTB (n)	4 [2; 12]	9 [3; 14]	12 [4; 29]	0 [1; 4]	3 [0; 6]	6 [0; 24]

PSV, Pressure Support Ventilation; NAVA, Neurally Adjusted Ventilatory Assist; RTB, reverse triggered breath. Data are shown as median (25th and 75th percentile).

## Data Availability

The authors will share all the individual participant data collected during the trial after de-identification, to researchers who provide a methodologically sound proposal. The full protocol and raw data are available at longhini.federico@gmail.com.
